# Methyl (2*E*)-2-cyano-3-(dimethyl­amino)­prop-2-enoate

**DOI:** 10.1107/S1600536812042304

**Published:** 2012-10-13

**Authors:** Rajni Kant, Vivek K. Gupta, Kamini Kapoor, D. R. Patil, D. K. Salunkhe, Madhukar B. Deshmukh

**Affiliations:** a X-ray Crystallography Laboratory, Post-Graduate Department of Physics & Electronics, University of Jammu, Jammu Tawi 180 006, India; b Department of Chemistry, Shivaji University, Kolhapur, 416 004, India

## Abstract

In the title compound, C_7_H_10_N_2_O_2_, the dimethyl­amino group is twisted slightly relative to the acrylate fragment, forming a dihedral angle of 11.6 (1)°. In the crystal, molecules are linked *via* pairs of bifurcated C—H/H⋯O hydrogen bonds, forming inversion dimers, which are further connected by C—H⋯N hydrogen bonds into chains along the *a-*axis direction.

## Related literature

For applications of enamines, see: Huang *et al.* (2007 ▶); Michael *et al.* (1999 ▶). For a related structure, see: Gupta *et al.* (2007 ▶).
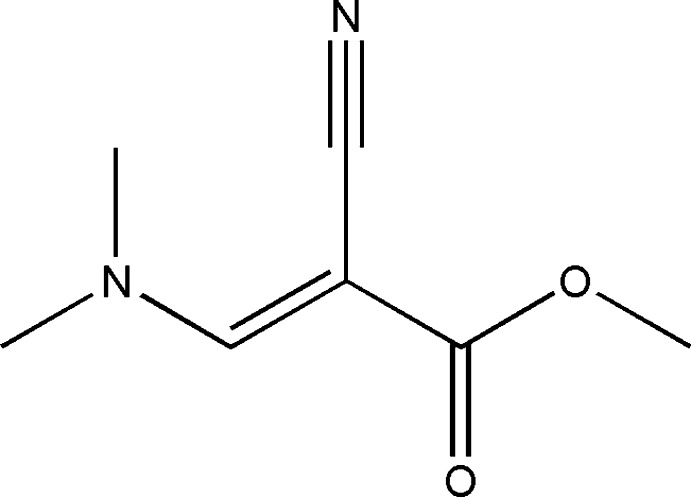


## Experimental

### 

#### Crystal data

C_7_H_10_N_2_O_2_
*M* *_r_* = 154.17
Triclinic, 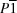

*a* = 7.1102 (5) Å
*b* = 7.8170 (5) Å
*c* = 8.2454 (6) Å
α = 97.270 (6)°
β = 93.431 (6)°
γ = 115.680 (7)°
*V* = 406.31 (5) Å^3^
*Z* = 2
Mo *K*α radiation
μ = 0.09 mm^−1^
*T* = 293 K
0.3 × 0.2 × 0.2 mm

#### Data collection

Oxford Diffraction Xcalibur Sapphire3 diffractometer
Absorption correction: multi-scan (*CrysAlis PRO*; Oxford Diffraction, 2010 ▶) *T*_min_ = 0.830, *T*_max_ = 1.000
6682 measured reflections
1593 independent reflections
1126 reflections with *I* > 2σ(*I*)
*R*_int_ = 0.047

#### Refinement

*R*[*F* ^2^ > 2σ(*F* ^2^)] = 0.046
*wR*(*F* ^2^) = 0.134
*S* = 1.03
1593 reflections
103 parameters
H-atom parameters constrained
Δρ_max_ = 0.17 e Å^−3^
Δρ_min_ = −0.13 e Å^−3^

### 

Data collection: *CrysAlis PRO* (Oxford Diffraction, 2010 ▶); cell refinement: *CrysAlis PRO*; data reduction: *CrysAlis PRO*; program(s) used to solve structure: *SHELXS97* (Sheldrick, 2008 ▶); program(s) used to refine structure: *SHELXL97* (Sheldrick, 2008 ▶); molecular graphics: *ORTEP-3* (Farrugia, 1997 ▶); software used to prepare material for publication: *PLATON* (Spek, 2009 ▶).

## Supplementary Material

Crystal structure: contains datablock(s) I, New_Global_Publ_Block. DOI: 10.1107/S1600536812042304/gk2525sup1.cif

Structure factors: contains datablock(s) I. DOI: 10.1107/S1600536812042304/gk2525Isup2.hkl

Supplementary material file. DOI: 10.1107/S1600536812042304/gk2525Isup3.cml

Additional supplementary materials:  crystallographic information; 3D view; checkCIF report

## Figures and Tables

**Table 1:** Hydrogen-bond geometry (Å, °)

*D*—H⋯*A*	*D*—H	H⋯*A*	*D*⋯*A*	*D*—H⋯*A*
C3—H3⋯O1^i^	0.93	2.56	3.370 (2)	146
C4—H4*A*⋯O1^i^	0.96	2.58	3.415 (3)	145
C7—H7*C*⋯N2^ii^	0.96	2.58	3.535 (3)	172

Symmetry codes: (i) 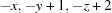; (ii) 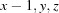.

**Fig. 1.**
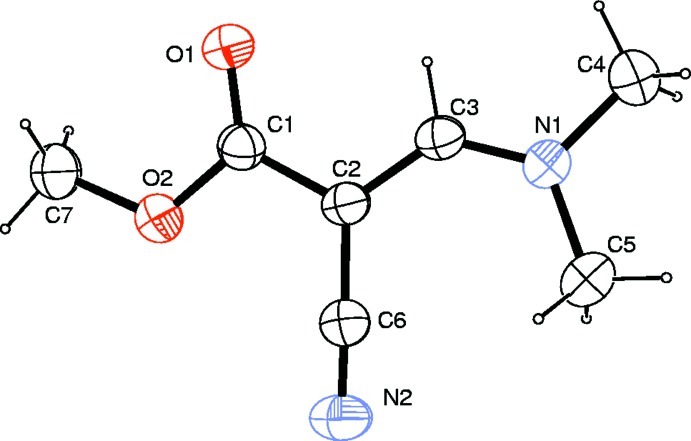
ORTEP view of the molecule with the atom-labeling scheme. The displacement ellipsoids are drawn at the 40% probability level. H atoms are shown as small spheres of arbitrary radii.

**Fig. 2.**
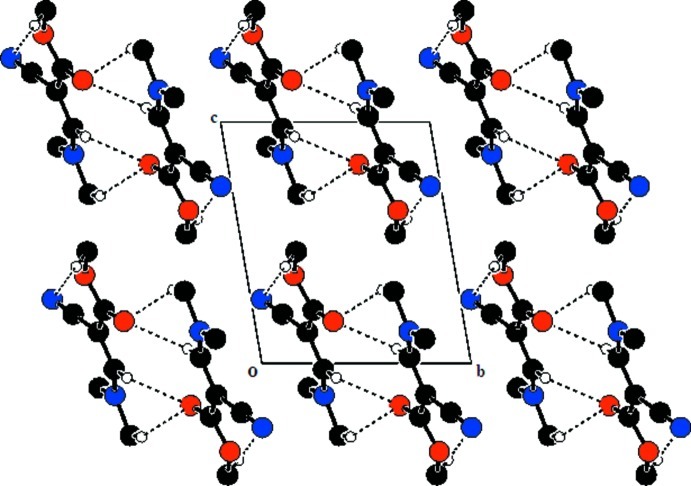
The packing arrangement of molecules viewed down the a axis. The dotted lines show intermolecular C—H···O and C—H···N hydrogen bonds.

## supplementary crystallographic information

### Comment

Enamines are the multipurpose synthetic intermediates used for the synthesis of a variety of organic derivatives, bioactive natural products and their analogs (Huang *et al.*, 2007; Michael *et al.*, 1999).

In (I)(Fig.1), all bond lengths and angles are normal and correspond to those observed in the related structure (Gupta *et al.*, 2007). The dihedral angle between dimethylamino and acrylate fragment is 11.6 (1)°. In the crystal, C3—H3···O1 and C4—H4A···O1 hydrogen bonds link molecules to form dimers. Dimers are further connected by C—H···N hydrogen bonds into chains along the *a* axis (Fig. 2, Table 1.).

### Experimental

In a 50 ml round bottom flask the mixture of 5 mmole of methyl cyanoacetate and 5 mmole of dimethylformamide dimethyl acetal was stirred at room temperature for 1–1.5 h. After completion of reaction, the reaction mixture was poured on ice cold water and the separated solid was precipated after 15 minutes and recrystallized from ethanol. Yield: 87%; m.p. 378–380 K. IR(KBr): 2205, 1695, 1621, 1433, 1373, 1287, 1216 1/cm

### Refinement

All H atoms were positioned geometrically and were treated as riding on their parent C atoms, with C—H distances of 0.93–0.96 Å and with *U*_iso_(H) = 1.2*U*_eq_(C) or 1.5*U*_eq_(methyl C).

### Crystal data

**Table:**

C_7_H_10_N_2_O_2_	*Z* = 2
*M**_r_* = 154.17	*F*(000) = 164
Triclinic, *P*1	*D*_x_ = 1.260 Mg m^−^^3^
Hall symbol: -P 1	Mo *K*α radiation, λ = 0.71073 Å
*a* = 7.1102 (5) Å	Cell parameters from 2720 reflections
*b* = 7.8170 (5) Å	θ = 3.5–29.0°
*c* = 8.2454 (6) Å	µ = 0.09 mm^−^^1^
α = 97.270 (6)°	*T* = 293 K
β = 93.431 (6)°	Block, white
γ = 115.680 (7)°	0.3 × 0.2 × 0.2 mm
*V* = 406.31 (5) Å^3^	

### Data collection

**Table:**

Oxford Diffraction Xcalibur Sapphire3 diffractometer	1593 independent reflections
Radiation source: fine-focus sealed tube	1126 reflections with *I* > 2σ(*I*)
Graphite monochromator	*R*_int_ = 0.047
Detector resolution: 16.1049 pixels mm^-1^	θ_max_ = 26.0°, θ_min_ = 3.5°
ω scan	*h* = −8→8
Absorption correction: multi-scan (*CrysAlis PRO*; Oxford Diffraction, 2010)	*k* = −9→9
*T*_min_ = 0.830, *T*_max_ = 1.000	*l* = −10→10
6682 measured reflections	

### Refinement

**Table:**

Refinement on *F*^2^	Primary atom site location: structure-invariant direct methods
Least-squares matrix: full	Secondary atom site location: difference Fourier map
*R*[*F*^2^ > 2σ(*F*^2^)] = 0.046	Hydrogen site location: inferred from neighbouring sites
*wR*(*F*^2^) = 0.134	H-atom parameters constrained
*S* = 1.03	*w* = 1/[σ^2^(*F*_o_^2^) + (0.0618*P*)^2^ + 0.0587*P*] where *P* = (*F*_o_^2^ + 2*F*_c_^2^)/3
1593 reflections	(Δ/σ)_max_ = 0.001
103 parameters	Δρ_max_ = 0.17 e Å^−^^3^
0 restraints	Δρ_min_ = −0.13 e Å^−^^3^

### Special details

**Table:**

Experimental. *CrysAlis PRO*, Oxford Diffraction Ltd., Version 1.171.34.40 (release 27–08-2010 CrysAlis171. NET) (compiled Aug 27 2010,11:50:40) Empirical absorption correction using spherical harmonics, implemented in SCALE3 ABSPACK scaling algorithm.
Geometry. All e.s.d.'s (except the e.s.d. in the dihedral angle between two l.s. planes) are estimated using the full covariance matrix. The cell e.s.d.'s are taken into account individually in the estimation of e.s.d.'s in distances, angles and torsion angles; correlations between e.s.d.'s in cell parameters are only used when they are defined by crystal symmetry. An approximate (isotropic) treatment of cell e.s.d.'s is used for estimating e.s.d.'s involving l.s. planes.
Refinement. Refinement of *F*^2^ against ALL reflections. The weighted *R*-factor *wR* and goodness of fit *S* are based on *F*^2^, conventional *R*-factors *R* are based on *F*, with *F* set to zero for negative *F*^2^. The threshold expression of *F*^2^ > σ(*F*^2^) is used only for calculating *R*-factors(gt) *etc*. and is not relevant to the choice of reflections for refinement. *R*-factors based on *F*^2^ are statistically about twice as large as those based on *F*, and *R*- factors based on ALL data will be even larger.

### Fractional atomic coordinates and isotropic or equivalent isotropic displacement parameters (Å^2^)

**Table:**

	*x*	*y*	*z*	*U*_iso_*/*U*_eq_	
O1	0.0160 (2)	0.6248 (2)	0.82511 (17)	0.0648 (5)	
O2	0.20619 (19)	0.77535 (19)	0.63425 (16)	0.0535 (4)	
C1	0.1860 (3)	0.7109 (2)	0.7792 (2)	0.0438 (4)	
C2	0.3885 (3)	0.7570 (2)	0.8695 (2)	0.0405 (4)	
N1	0.5414 (2)	0.7293 (2)	1.13299 (19)	0.0475 (4)	
N2	0.7150 (3)	0.9493 (3)	0.7343 (2)	0.0733 (6)	
C3	0.3876 (3)	0.7106 (2)	1.0252 (2)	0.0423 (4)	
H3	0.2552	0.6567	1.0599	0.051*	
C4	0.5009 (3)	0.6883 (3)	1.2987 (3)	0.0666 (6)	
H4A	0.3525	0.6365	1.3051	0.100*	
H4B	0.5737	0.8051	1.3773	0.100*	
H4C	0.5498	0.5964	1.3228	0.100*	
C5	0.7594 (3)	0.7984 (3)	1.1018 (3)	0.0616 (6)	
H5A	0.7642	0.7414	0.9929	0.092*	
H5B	0.8346	0.7633	1.1813	0.092*	
H5C	0.8230	0.9360	1.1107	0.092*	
C6	0.5718 (3)	0.8620 (3)	0.7958 (2)	0.0478 (5)	
C7	0.0158 (3)	0.7445 (3)	0.5374 (3)	0.0611 (6)	
H7A	−0.0765	0.6090	0.5118	0.092*	
H7B	0.0491	0.7950	0.4371	0.092*	
H7C	−0.0524	0.8090	0.5988	0.092*	

### Atomic displacement parameters (Å^2^)

**Table:**

	*U*^11^	*U*^22^	*U*^33^	*U*^12^	*U*^13^	*U*^23^
O1	0.0394 (8)	0.0845 (10)	0.0603 (9)	0.0136 (7)	0.0073 (7)	0.0287 (8)
O2	0.0476 (8)	0.0646 (9)	0.0472 (8)	0.0215 (6)	0.0059 (6)	0.0186 (6)
C1	0.0443 (11)	0.0417 (9)	0.0417 (10)	0.0157 (8)	0.0064 (8)	0.0071 (7)
C2	0.0356 (9)	0.0396 (9)	0.0435 (10)	0.0140 (7)	0.0079 (8)	0.0061 (7)
N1	0.0395 (8)	0.0516 (9)	0.0457 (9)	0.0151 (7)	0.0027 (7)	0.0095 (7)
N2	0.0502 (11)	0.0953 (15)	0.0766 (13)	0.0263 (10)	0.0241 (10)	0.0380 (11)
C3	0.0369 (10)	0.0385 (9)	0.0467 (10)	0.0122 (8)	0.0069 (8)	0.0073 (7)
C4	0.0583 (13)	0.0804 (15)	0.0516 (12)	0.0205 (11)	0.0027 (10)	0.0200 (11)
C5	0.0422 (11)	0.0738 (14)	0.0650 (14)	0.0223 (10)	0.0028 (10)	0.0140 (11)
C6	0.0430 (10)	0.0534 (11)	0.0475 (11)	0.0210 (9)	0.0086 (9)	0.0114 (9)
C7	0.0567 (13)	0.0726 (14)	0.0569 (13)	0.0298 (11)	0.0024 (10)	0.0200 (11)

### Geometric parameters (Å, º)

**Table:**

O1—C1	1.213 (2)	C3—H3	0.9300
O2—C1	1.346 (2)	C4—H4A	0.9600
O2—C7	1.438 (2)	C4—H4B	0.9600
C1—C2	1.454 (2)	C4—H4C	0.9600
C2—C3	1.377 (2)	C5—H5A	0.9600
C2—C6	1.427 (2)	C5—H5B	0.9600
N1—C3	1.311 (2)	C5—H5C	0.9600
N1—C5	1.455 (2)	C7—H7A	0.9600
N1—C4	1.460 (2)	C7—H7B	0.9600
N2—C6	1.143 (2)	C7—H7C	0.9600
			
C1—O2—C7	116.82 (15)	N1—C4—H4C	109.5
O1—C1—O2	122.40 (17)	H4A—C4—H4C	109.5
O1—C1—C2	125.53 (17)	H4B—C4—H4C	109.5
O2—C1—C2	112.07 (15)	N1—C5—H5A	109.5
C3—C2—C6	125.51 (17)	N1—C5—H5B	109.5
C3—C2—C1	117.00 (15)	H5A—C5—H5B	109.5
C6—C2—C1	117.21 (16)	N1—C5—H5C	109.5
C3—N1—C5	124.10 (16)	H5A—C5—H5C	109.5
C3—N1—C4	120.07 (15)	H5B—C5—H5C	109.5
C5—N1—C4	115.77 (16)	N2—C6—C2	177.6 (2)
N1—C3—C2	131.07 (16)	O2—C7—H7A	109.5
N1—C3—H3	114.5	O2—C7—H7B	109.5
C2—C3—H3	114.5	H7A—C7—H7B	109.5
N1—C4—H4A	109.5	O2—C7—H7C	109.5
N1—C4—H4B	109.5	H7A—C7—H7C	109.5
H4A—C4—H4B	109.5	H7B—C7—H7C	109.5
			
C7—O2—C1—O1	2.5 (3)	O2—C1—C2—C6	0.9 (2)
C7—O2—C1—C2	−177.56 (15)	C5—N1—C3—C2	−4.1 (3)
O1—C1—C2—C3	−4.8 (3)	C4—N1—C3—C2	173.00 (19)
O2—C1—C2—C3	175.27 (15)	C6—C2—C3—N1	−8.1 (3)
O1—C1—C2—C6	−179.14 (17)	C1—C2—C3—N1	178.06 (17)

### Hydrogen-bond geometry (Å, º)

**Table:**

*D*—H···*A*	*D*—H	H···*A*	*D*···*A*	*D*—H···*A*
C3—H3···O1^i^	0.93	2.56	3.370 (2)	146
C4—H4*A*···O1^i^	0.96	2.58	3.415 (3)	145
C7—H7*C*···N2^ii^	0.96	2.58	3.535 (3)	172

Symmetry codes: (i) −*x*, −*y*+1, −*z*+2; (ii) *x*−1, *y*, *z*.
